# Obstacles to Testing Molyneux’s Question Empirically

**DOI:** 10.1177/2041669515599330

**Published:** 2015-08-31

**Authors:** Tony Cheng

**Affiliations:** Department of Philosophy, University College London, UK

**Keywords:** Molyneux question, blind subjects, movement, raised-line drawings, amodal representation, touch

## Abstract

There have recently been various empirical attempts to answer Molyneux’s question, for example, the experiments undertaken by the Held group. These studies, though intricate, have encountered some objections, for instance, from Schwenkler, who proposes two ways of improving the experiments. One is “to re-run [the] experiment with the stimulus objects made to move, and/or the subjects moved or permitted to move with respect to them” (p. 94), which would promote three dimensional or otherwise viewpoint-invariant representations. The other is “to use geometrically simpler shapes, such as the cube and sphere in Molyneux’s original proposal, or planar figures instead of three-dimensional solids” (p. 188). Connolly argues against the first modification but agrees with the second. In this article, I argue that the second modification is also problematic (though still surmountable), and that both Schwenkler and Connolly are too optimistic about the prospect of addressing Molyneux’s question empirically.

## State of Play

William Molyneux, a Dublin Lawyer, once wrote to English philosopher John Locke (1960/1975) posing a thorny question (p. II/ix/8): Consider a congenitally blind person who is otherwise the same as a typically sighted person.

Suppose him now to gain the use of his sight. And suppose him to be presented with a cube and a sphere, of nighly the same bigness. Quaere, will he be able to tell, by the use of his vision alone, which is the sphere, and which is the cube?

Although this sudden restoration of sight involving no other concomitant issues may not be empirically realistic, several philosophers, and scientists have attempted to treat Molyneux’s question empirically; Held (2009) and Held et al. (2011) being prominent recent attempts. Schwenkler (2012, 2013) has argued that these studies do not adequately address Molyneux’s question because the participants “could not form the requisite visual representations of those shapes in the first place” (2013, p. 92). Held and colleagues did demonstrate that the participants had visual representations sufficient for performing visual matching, but Schwenkler argues that these representations could have been based on viewpoint-dependent or low-level features, such as number of lines, and proposes two ways to fix this problem. Connolly (2013) criticizes one of these proposed solutions but endorses the other. In what follows I attempt to show that this second solution, though not irremediable, is nevertheless problematic as it stands.

## A Case for Pessimism

Here is a brief description of the Held experiment we will be focusing on. It involves five newly sighted subjects with 20 pairs of objects as stimuli, constructed from Lego blocks. The experimenters have ensured that the stimuli “are large enough … to sidestep any acuity limitations of the subjects” (Held et al., 2011, p. 551). The task involves two steps. First, the participants are presented with one target stimulus, either tactilely or visually depending on the conditions (see later). Then, two further stimuli are shown, again either tactilely or visually. One of the latter stimuli will be identical to the target stimulus. The task is to identify which one that is. There are three conditions in the experiment: touch-to-touch (TT), vision-to-vision (VV), and touch-to-vision (TV) tasks. The crucial question is whether recognition of the original shape is worse in the TV task than the other two.

It turned out that participants had high performances in both the TT task (98% accuracy) and the VV task (92% accuracy). However, they did poorly in the crucial TV task (58% accuracy). After five days and without explicit training, the participants improved from 58% to circa 80% accuracy in the TV task. Based on their results, Held et al. (2011) cautiously suggested “that the answer to Molyneux’s Question is likely negative” (p. 552). The basic logic is this: given that the participants had high performances in both the TT and VV tasks, how well they did in the TV task indicates what we should say about Molyneux’s question. Given the poor performances (58% accuracy), the Held et al. conclusion seems to be warranted. Schwenkler’s argument is that there is a sensible alternative explanation of the high performances in the VV task that has not been ruled out, that is, the high accuracy in the VV task does not show that the participants already have good enough visual capacity required by Molyneux’s question.

For the sake of argument,^1^ let’s agree with Schwenkler’s criticism of the studies performed by Held et al. (2013):

In the VV task, subjects needed only to make gross discriminations based on the overall appearance of the stimuli, which were presented from a single viewing angle. Intuitively, this can be done by attending to low-level visual features like colour, shadow and approximate overall contours. (p. 91)

However, I share Connolly’s “worry about whether subjects can appreciate depth cues immediately post-surgery” (2013, p. 510). Given this worry, the plausibility of Schwenkler’s first suggestion, that we should “re-run [the] experiment with the stimulus objects made to move, and/or the subjects moved or permitted to move with respect to them” (2013, p. 94), is indeed weakened. Moreover, depth from motion (motion parallax) is only one of at least 13 cues used by the visual system to infer relative and absolute positions in-depth (Palmer, 1999, p. 204). These include cues from the eyes, such as stereopsis and lens focus (accommodation), dynamic information from movement of the viewer relative to the object, such as motion parallax and texture accretion or deletion, and monocular cues present in static images, such as occlusion, aerial perspective, shading, shadows, blur, familiar size, and so on. It is, therefore, unclear why motion parallax, rather than any other depth cue, was singled out as necessary to provide newly sighted individuals with depth information.

What I want to focus on here is Connolly’s elaboration of the second suggestion offered by Schwenkler, namely that running the experiment with two-dimensional (2D) stimuli can fix the problem because newly sighted individuals may not be capable of forming a three-dimensional (3D) representation of viewed objects. Connolly invokes raised-line drawings to make his point. These types of drawings are used to make graphical, typically 2D printed materials, accessible to individuals who are visually impaired or blind. Connolly’s proposal seems plausible if the Molyneux experiment were to be run in 2D: Raised-line drawings are exactly the kind of stimuli blind people encounter in the 2D context. Of course, it is not *really* 2D: Raised-line drawings are *raised* so that a distinction between the object and its background can be made, but the third dimension here is not relevant for object recognition: If the raised-line drawing in question stands for a pair of scissors, to change how much we raise the lines does not affect what is represented, though it might affect the difficulty of the task. Connolly’s suggestion here is not lacking its intelligibility. Some vision scientists might be puzzled by this suggestion, since the most interesting point in this area is whether the blind subjects have relevant 3D representations. However, the original Molyneux’s question concerns whether there is *any* shared amodal spatial representation between sight and touch, so given this purpose 2D stimuli are suffice for relevant experiments.^2^

However, for a number of reasons, this modification still might not work. As all parties agree, in vision the formation of 3D object representations constructed from 2D projections of the object onto the retina is a computationally difficult process, which may not be functional in newly sighted individuals. These patients would need experience or training to use their vision to form 3D representations of objects and their environment. Patient SB studied by Gregory and Wallace (1963) was able to read the time on a clock, but was *not* able to judge distance. This case is not ideal as evidence here as SB did have residual vision in his first year, but at least this case chimes well with the current point.^3^ This contrasts with the visual recognition of 2D depictions, such as line drawings and photographs, which can be matched to 3D objects with no experience or training with 2D depictions (Hochberg & Brooks, 1962; Kennedy & Ross, 1975). *Interestingly, in the tactile domain, the difficulty is reversed*: Even for those who are very familiar with identifying objects by touch, such as blind subjects, it is very difficult for them to learn how to associate those objects they encountered in 3D context with touch with their depictions in 2D raised-line drawings. Because of this, the proposal involving raised-line drawings is problematic: One wants to use 2D stimuli because, with regard to vision, going from 2D to 3D is likely to be difficult for newly sighted subjects; *but similarly, and crucially, in terms of touch moving from 3D to 2D is difficult*, perhaps even more so for currently or formerly blind subjects, who lack experience with line-based depictions that are common with vision (Heller, 1989). Therefore in moving from 3D to 2D, we fix one problem (that of generating 3D representations from 2D retinal projections in vision) but generate another (that of generating 2D representations from 3D objects in touch).

Connolly preempts this objection to some extent:

[A]s Picard and Lebaz summarize, identifying raised-line drawings by touch is “hard but not impossible” (Picard and Lebaz, 2012, p. 427). … All of this suggests that rather than indicating that a two-dimensional test for Molyneux’s question is a non-starter, the raised-line drawing studies point to constraints for such a test instead. (Connolly, 2013, p. 509)

I agree with Connolly that this task is not *impossible*: Perhaps more intensive training for blind subjects with raised-line drawings before undergoing the relevant surgery and subsequent experiment would do. But I want to focus on the *obstacles* for this improvement: In the same paragraph, Connolly suggests that the reason why object-recognitions tasks involving raised-line drawings are so difficult is because often the objects in question are too complex. For the purpose of running the Molyneux experiment, using simple shapes such as circles and squares is sufficient (Connolly, 2013, p. 509). However, most blind children find that even with these simple shapes the task is very difficult, and make many mistakes. There could be many reasons for this, but one is that in the real world blind children are limited in terms of the amount of things they touch, for both safety and convenience reasons, and because teachers and caregivers do not have the time to generate as many tactile examples for their blind students as there are visual examples of objects in both 3D and 2D. This generates two problems: First, their experience of encountering different kinds of things is very limited; Second, and more importantly, their concepts of shape might be different from those of typically sighted people: For example, it is not obvious for many blind children whether continuity of contours is necessary for the integrity of shapes. This should not surprise us, since in Evans (1985) among others, we have seen that Molyneux’s question is not purely perceptual, but also involves *conceptual* capacities. To be sure, this difficulty can be remedied empirically: Those subjects just need more extensive training. But I want to emphasise that the difficulties are more serious than some researchers might have supposed.^4^

## Looking Ahead

The conclusion to be drawn is this: Molyneux’s question, at least formulated in a certain way, is indeed an empirical question, but the empirical obstacles are more challenging than many have supposed. To say that a question is empirically testable is to say that the very idea of the relevant experiment does not violate empirical laws—such as physical laws. In Molyneux’s statement of the question, as quoted at the beginning of the article, there is *no* clash with empirical laws. I said that “the sudden restoration of sight with no other concomitant issues may not be empirically *realistic*,” but it is still empirically *possible* to test it. In this regard, I agree with Held et al., Schwenkler, and Connolly. I also believe that the suggested modification concerning 2D stimuli is a constructive contribution to the discussion. However, Schwenkler and Connolly seem to think that, with the minimal adjustments they propose, the experiment would be ready to go. I hope to have shown that given blind subjects’ limited experiences even with tactile objects, although it is still empirically possible to test Molyneux’s question, the obstacles are more challenging than Schwenkler and Connolly imply.

## Notes

1 In particular, it is unclear why Molyneux’s question does not allow for the mapping between sight and touch to be based on low-level features too, but due to restrictions of space, I do not elaborate on this point here.

2 I am grateful to Michael Morgan for pointing this out.

3 Again Michael Morgan points this out.

4 A different problem for using 2D stimuli is raised by Morgan (1977/2008): In a nutshell, if the stimuli are 2D, the matching might be achieved at the *cognitive* level. The 3D stimuli in this regard can ensure that the performances are *perceptual* because, for example, a sphere is uniform in a way that a cube is not. This problem, if genuine, is independent of the concern I have tried to elaborate here.
